# Exploring the biomarkers and potential therapeutic drugs for sepsis via integrated bioinformatic analysis

**DOI:** 10.1186/s12879-023-08883-9

**Published:** 2024-01-02

**Authors:** Pingping Liang, Yongjian Wu, Siying Qu, Muhammad Younis, Wei Wang, Zhilong Wu, Xi Huang

**Affiliations:** 1 Foshan Fourth People’s Hospital, Guangdong Province, Foshan, 528041 China; 2 Center for Infection and Immunity and Guangdong Provincial Engineering Research Center of Molecular Imaging, the Fifth Affiliated Hospital of Sun Yat-Sen University, Guangdong Province, Zhuhai, 519000 China; 3 Department of Clinical Laboratory, Zhuhai Hospital of Integrated Traditional Chinese and Western Medicine, The Second People’s Hospital of Zhuhai, Guangdong Province, Zhuhai, 519020 China

**Keywords:** Sepsis, Biomarkers, Integrated transcriptome, Therapy, Drugs

## Abstract

**Background:**

Sepsis is a life-threatening condition caused by an excessive inflammatory response to an infection, associated with high mortality. However, the regulatory mechanism of sepsis remains unclear.

**Results:**

In this study, bioinformatics analysis revealed the novel key biomarkers associated with sepsis and potential regulators. Three public datasets (GSE28750, GSE57065 and GSE95233) were employed to recognize the differentially expressed genes (DEGs). Taking the intersection of DEGs from these three datasets, GO and KEGG pathway enrichment analysis revealed 537 shared DEGs and their biological functions and pathways. These genes were mainly enriched in T cell activation, differentiation, lymphocyte differentiation, mononuclear cell differentiation, and regulation of T cell activation based on GO analysis. Further, pathway enrichment analysis revealed that these DEGs were significantly enriched in Th1, Th2 and Th17 cell differentiation. Additionally, five hub immune-related genes (*CD3E*, *HLA-DRA*, *IL2RB*, *ITK* and *LAT*) were identified from the protein–protein interaction network, and sepsis patients with higher expression of hub genes had a better prognosis. Besides, 14 drugs targeting these five hub related genes were revealed on the basis of the DrugBank database, which proved advantageous for treating immune-related diseases.

**Conclusions:**

These results strengthen the new understanding of sepsis development and provide a fresh perspective into discriminating the candidate biomarkers for predicting sepsis as well as identifying new drugs for treating sepsis.

**Supplementary Information:**

The online version contains supplementary material available at 10.1186/s12879-023-08883-9.

## Background

Sepsis is a fatal organ dysfunction produced by a dysregulated response to infection [1]. Sepsis is responsible for an increasing number of deaths worldwide each year [2]. According to the World Health Organization, sepsis causes around 6 million deaths worldwide each year and the majority of which can be prevented [3]. The World Health Organization adopted a resolution in 2017 to enhance sepsis prevention, detection, and treatment, recognizing it as a global health priority [4]. According to a newly published comprehensive review, among all patients treated for sepsis in hospital, HA (hospital acquired) sepsis accounted for 23.6% of cases, with a 95% confidence interval of 17% to 31.8%, and a range spanning from 16% to 36.4%. Within the intensive care unit (ICU), 24.4% of sepsis cases involving organ dysfunction occurred during the patient’s stay in the ICU, and this estimate had a 95% confidence interval of 16.7% to 34.2%, along with a range from 10.3% to 42.5%. Additionally, nearly half of all cases of sepsis, specifically 48.7%, originated within the hospital, with a 95% confidence interval of 38.3% to 59.3%, and a range extending from 18.7% to 69.4% [5]. The complicated host immune response during and after sepsis includes an early, excessively inflammatory reaction of the host in response to infection resulting in tissue damage, organ failure, and impaired endothelial function [6]. However, decades of attempts to reduce the harmful effects of this excessive inflammation through anti-inflammatory treatment methods have failed, which prompting medical professionals and academics to reevaluate the biology of sepsis [7]. Treatment for sepsis remains largely supportive, with simple measures; we still don't have a single treatment that can consistently save the lives of patients with sepsis [8–10]. Therefore, it is better to further elucidate the mechanism of sepsis to find more efficient drugs for effective and precise treatment reducing unnecessary costs, mortality and complications.

The use of biomarkers is extremely important for identifying, diagnosing, therapy, following up, stratification and predicting outcomes of diseases like sepsis. Biomarkers can provide important information because they can indicate the severity of sepsis, guide the clinicians to rapid diagnosis and treatment beyond the standard therapy and provide ongoing information on disease activity [11]. A wide range of sepsis biomarkers (cytokines, cell membrane receptors, metabolites, chemokines, cell proteins, complement component system etc.) has been described. However, their effectiveness in many instances is limited by insufficient specificity or sensitivity [12]. In addition, no single biomarker has been found to have sufficient diagnostic power to be used as a standard diagnostic tool. Consequently, there is a need to search for effective biomarkers in sepsis to improve early diagnosis, monitor therapeutic efficacy and improve prognosis.

Early diagnosis is crucial for prompt treatment, enhancing sepsis outcomes [13]. Delaying sepsis treatment increases the chance of mortality [14]. Sepsis is a highly heterogeneous syndrome with complex pathophysiology, excessive inflammation and immunosuppression [9]. The occurrence and progression of sepsis are significantly influenced by the immunological response of immune cells, such as T cells, NK cells, macrophages, and others [9]. In sepsis, severe lymphopenia and apoptosis of lymphocytes may be a significant cause of death [15]. Therefore, the differences in molecular expression patterns linked to immunological and inflammatory pathways require deep consideration and rigorous research.

Infection prevention is the only way to prevent sepsis, and vaccines are an important tool in reducing the risk of infections. Vaccines work by imitating the viral infection, causing the body to produce t-lymphocytes and antibodies that can recognize and destroy the invading organism [16, 17]. Vaccination can be an effective way to prevent infections that can lead to sepsis. Many infections that can lead to sepsis are becoming resistant to antibiotics, so preventing them by vaccination is becoming increasingly important [17, 18].

Despite advancements in the last ten years in describing sepsis-induced immunological dysfunctions, many unanswered concerns still exist, and several issues require additional studies [1]. Since high-throughput technologies generate large amounts of data, there is a need for effective bioinformatics tools that enable us to comprehend how molecules interact and control the various biological processes of health and disease [19–21]. The molecular understanding of sepsis has opened a new chapter because of transcriptome-based research. The transcriptome is the collection of all RNA molecules transcribed by the genome of a specific cell at a specific physiological or pathological condition [22, 23]. Each of these specific molecules presents a different functional spectrum in the cell and responds differently to environmental stimuli [24, 25]. By analyzing the gene expression, researchers can explore the molecular basis of sepsis in multiple ways and provide information about sepsis progression, as well as the discovery of new and previously unknown biomarkers.

Consequently, it is very important and urgent to explore the genetic changes that occur during the pathogenesis of sepsis. Finding a biomarker and panel of biomarkers could be a new avenue to provide new approaches to treat sepsis. Based on the above-discussed facts, the current research aims to explore the genetic changes associated with disease development and understand the pathophysiology of sepsis to find novel biomarkers and candidate drugs that may be useful for sepsis therapy. We hope these biomarkers will reveal important insights and impact on sepsis treatment.

## Materials and methods

### Data acquisition and processing

Three microarray datasets (GSE28750 [26], GSE57065 [27] and GSE95233 [28]) which made by the same platform were downloaded from the GEO database. Immune-related genes were extracted from the ImmPort website [29]. The immune-related gene sets contained 1793 genes after removing the duplicated genes. The detail is shown in Supplementary Table 1.

When multiple probes correspond to a gene, the average value is taken. When the probe had no corresponding gene symbol, the probe was eliminated. The matrix data for each GEO dataset was normalized and converted to base-2 logarithms using the R software’s "limma" (version 3.54.2) function [30].

### Differential expression analysis or Identification of shared DEGs among different microarray datasets

Differential expression analysis was conducted separately by the “limma” package [30] for GSE28750, GSE57065 and GSE95233 datasets. DEGs were genes with an adjusted P-value of 0.05 and |log2 fold change (log2FC)|> 1. DEGs found in each microarray dataset were shown with a volcano plot by the Enhancevolcano package (version 1.16.0) in R, and the common DEGs among these three datasets were shown with a Venn plot. Common DEGs with the same expression trends in each dataset were further selected.

To obtain genes related to immune and sepsis, the intersection of common DEGs obtained from three microarray datasets and genes in the immune-related gene set were taken using the Venn Diagram package (version 1.7.3) in R.

### DEGs enrichment analysis

The potential function of target genes was performed by GO and KEGG [31–33] enrichment analysis through clusterProfiler (version 4.7.1.003) [34] in R. Adjusted *P*-value < 0.05 was taken into account as statistically significant.

### PPI network construction and module analysis

The PPI network of immune-related DEGs was constructed by STRING [35] with an interaction score > 0.7. The PPI network was visualized using Cytoscape (version 3.8.2) [36], and core functional modules were analyzed using the plugin Molecular Complex Detection (MCODE) with default settings.

### Hub genes identification and analysis

Using the Cytoscape plugin cytoHubba to screen hub genes, then 12 topological algorithms (BottleNeck, Closeness, Betweenness, ClusteringCoefficient, Degree, DMNC, EPC, EcCentricity, MCC, MNC, Radiality and Stress) were exploited to confirm the final hub genes, which were visualized by UpSetR package (version 1.4.0). Subsequently, GeneMANIA [37] was exploited to construct an identified hub gene co-expression network.

### ROC curves and KM survival curves of hub genes

The hub genes identified were confirmed in GSE54514 and GSE65682. ROC curves were constructed and the area under the ROC curve (AUC) was calculated respectively, and R package “pROC” (version 1.18.4) [38] was used to measure the diagnostic capability of the hub genes. The best cutoff performed the classification of patients into high or low expression levels of hub genes. The difference between the high and the low survival curves was assessed using a log-rank test.

### Drugs from the DrugBank

Drugs targeting hub genes were retrieved from the DrugBank database [39]. Drugs including investigational drugs, FDA-approved drugs, experimental drugs, etc.

### Preparation of peripheral blood samples

Peripheral blood mononuclear cells (PBMCs) were obtained from heparin-anticoagulated fresh blood samples by density gradient centrifugation. After the collection of 2mL of EDTA-anticoagulated blood, we immediately isolated peripheral blood mononuclear with lymphocyte separation medium (Beyotime).

### Flow cytometry analysis

For surface staining, cells were washed and stained for 30 min with fluorescently conjugated monoclonal antibodies (mAbs). The mAbs of human targets for flow cytometry were as follows: CD4-APC (300,514, BioLegend), CD3E-FITC (300,405, BioLegend), HLA-DR-PE (307,605, BioLegend), CD25 (IL-2R)-PEcy7 (302,611, BioLegend). After staining, cells were washed in cold phosphate-buffered saline (PBS).

### Real‑Time quantitative Polymerase Chain Reaction (RT‑qPCR)

Total RNA was isolated with TRIzol reagent (Thermo Fisher Scientific). First-strand complementary DNA (cDNA) was obtained with a Synthesis Kit (Thermo Fisher Scientific); 2 μL of total cDNA and Synergy Brands (SYBR) Green PCR Master Mix (Applied Biosystems) were mixed. Then Eppendorf Master Cycle Realplex2 was used for real-time PCR (40 cycles). The RT-qPCR conditions were as follows: 3 min of enzyme activation at 95 °C, followed by denaturation at 95 °C for 20 s and annealing of primers at 60 °C for 20 s, and extension at 72 °C for 20 s. qPCR data were analyzed by the ΔΔCT method.

### Statistical analysis

All analyses were performed in R software. Wilcox test was used to compare the significant levels of hub genes in validated datasets. Significance was defined as p < 0.05.

## Results

### Identification of DEGs and common DEGs among three microarray datasets of sepsis

**Fig. 1:**
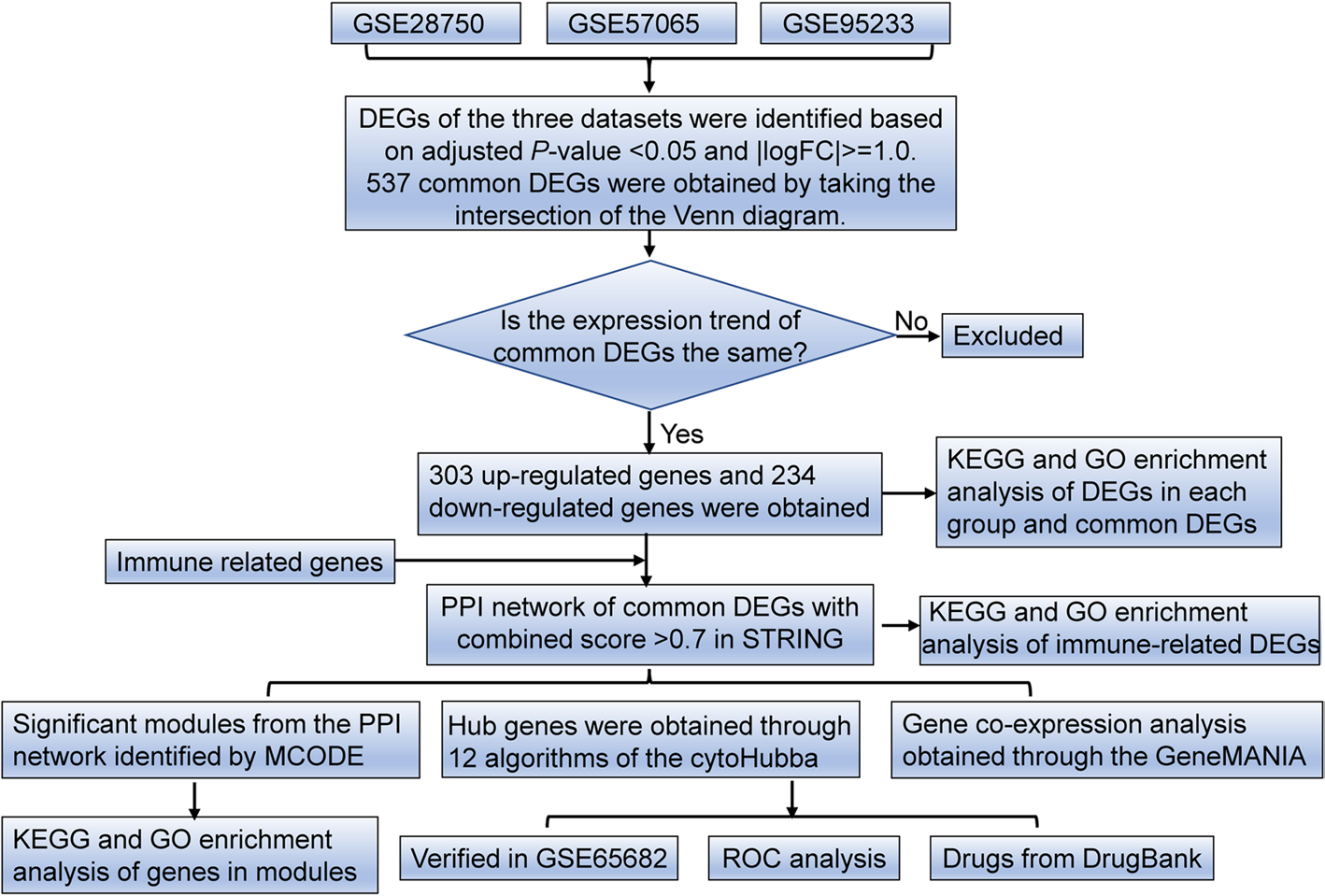
The flowchart for this research

**Fig. 2:**
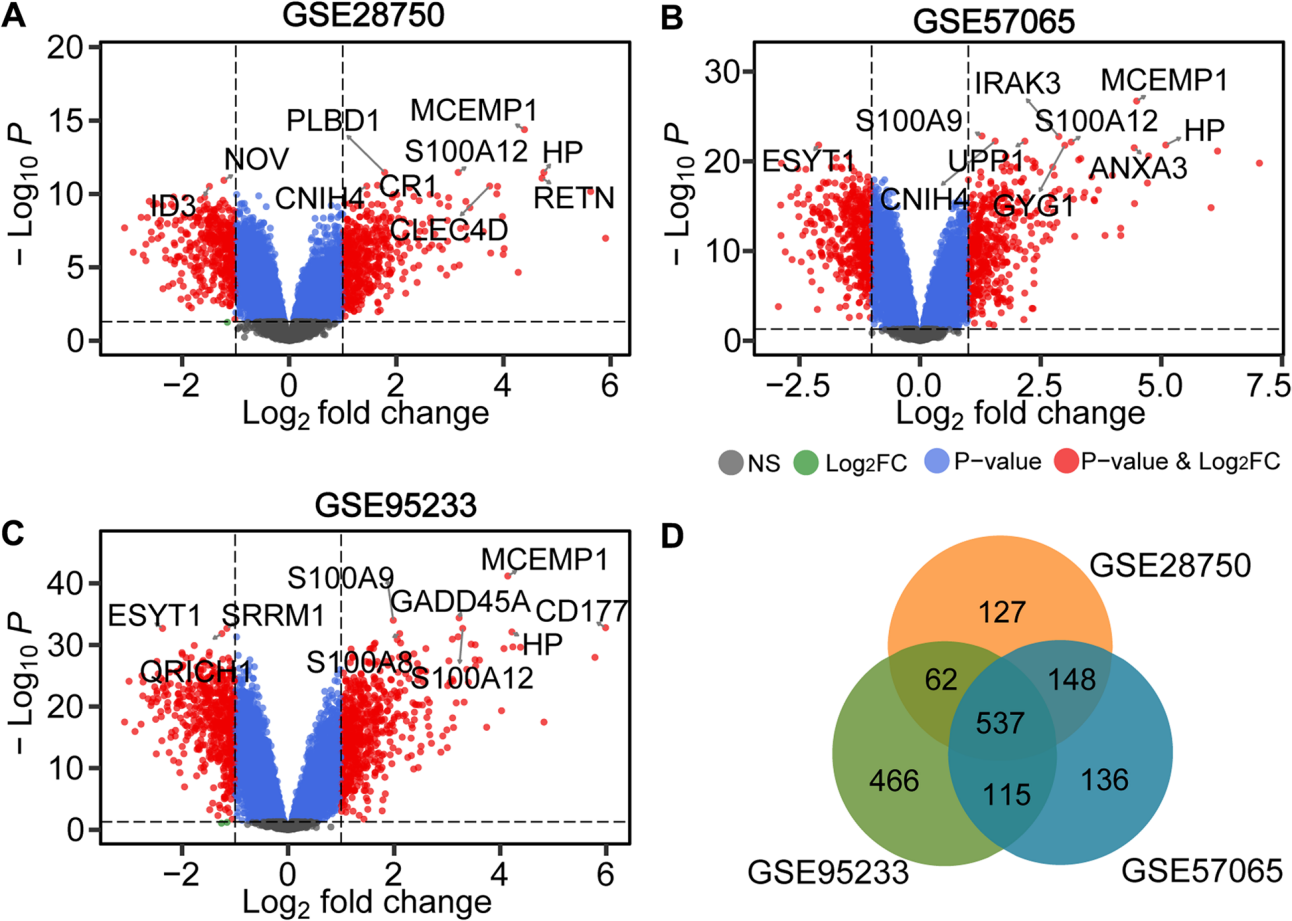
Volcano plots and Venn diagram of DEGs in sepsis and healthy at three datasets. **A**-**C** The volcano plots of DEGs in datasets of GSE28750, GSE57065 and GSE95233, red circles indicate significant DEGs (FDR *p*-value < 0.05) with minimum absolute fold change = 2. **D** The Venn diagram exhibited an overlap of 537 DEGs in these three datasets

The overall flow chart of this study was shown in Fig. 1. To explore the differentially expressed genes in sepsis, three public datasets (GSE28750, GSE57065 and GSE95233) were used. For the GSE28750 dataset, 403 down-regulated genes and 471 up-regulated genes were identified (Fig. 2A). The GSE57065 dataset showed 936 DEGs, including 485 up-regulated genes and 451 downregulated genes (Fig. 2B). For the GSE95233 dataset, a total of 1180 DEGs were obtained, among which 544 were down-regulated and 636 were up-regulated (Fig. 2C). Then, taking the intersection of DEGs from three datasets, there were 537 shared DEGs with consistent expression trends (Fig. 2D). The top significant DEGs among three datasets were *MCEMP1*, *HP*, *S100A12*, *ANXA3*, *HK3*, *CD177*, *GPR84*, *UPP1*, *GYG1*, and so on (Supplementary Table 2).

### GO and KEGG pathway enrichment analysis

**Fig. 3:**
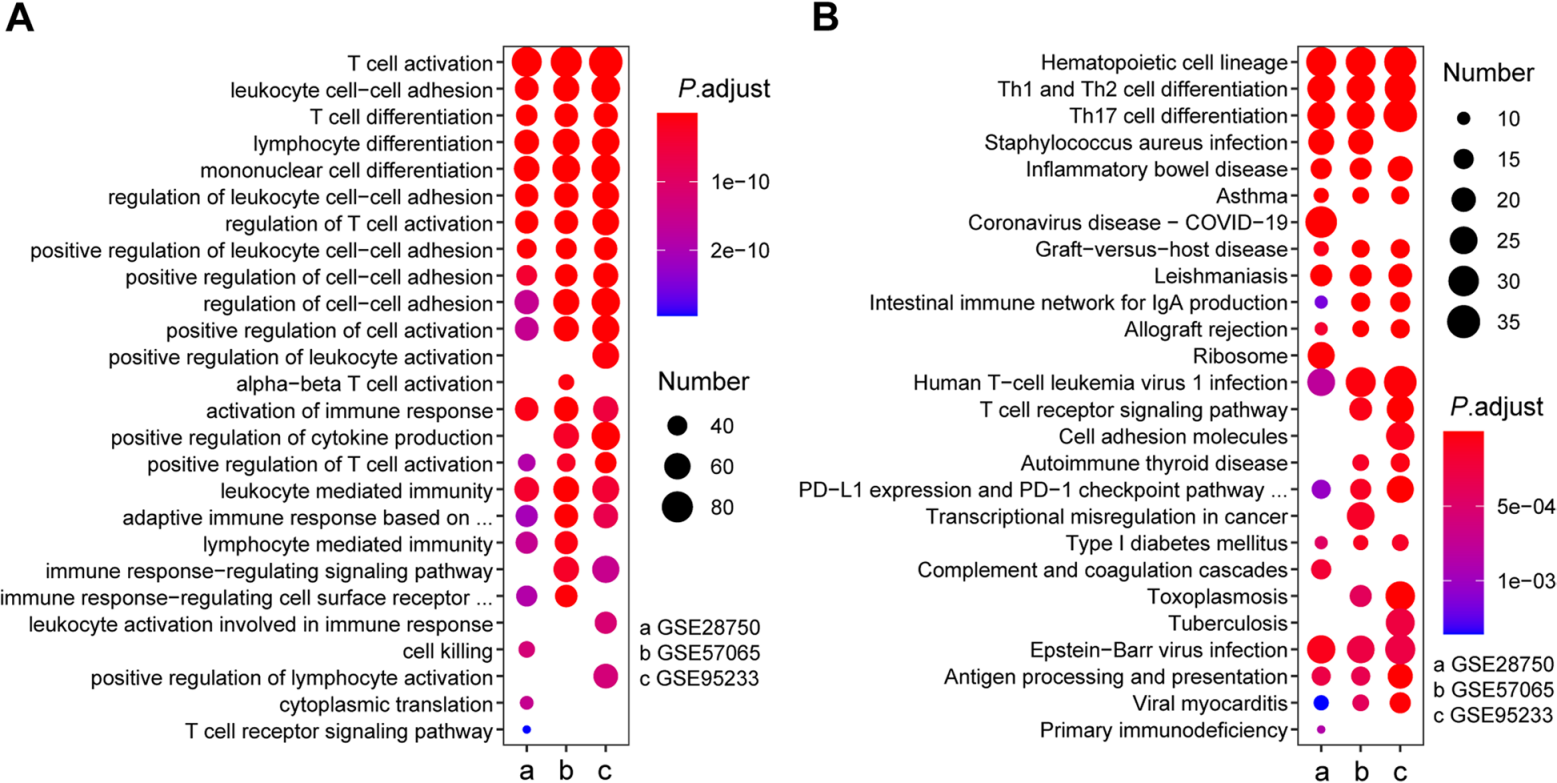
Enrichment analysis results of DEGs identified in the three datasets. **A** Top 20 GO terms in the biological process of DEGs identified in each dataset (a: GSE28750; b: GSE57065; c: GSE95233). **B** Top 20 KEGG pathways of DEGs identified in each dataset (a: GSE28750; b: GSE57065; c: GSE95233)

We performed the GO and KEGG pathway enrichment analysis to analyze the biological functions and pathways of each group (sepsis *vs* healthy controls) and 537 common DEGs. For each group of GO analysis, we selected the top 20 GO terms in the biological process, and there were 15 GO terms shared in all datasets, such as T cell differentiation, leukocyte cell–cell adhesion, T cell activation, and leukocyte differentiation (Fig. 3A). In addition, there were also 15 KEGG pathways shared in all datasets, such as hematopoietic cell lineage, Staphylococcus aureus infection, Th1 and Th2 cell differentiation, Th17 cell differentiation, etc*.* (Fig. 3B) [31–33]. As for common DEGs among three datasets, these genes were mainly enriched in T cell differentiation (*P* = 2.64e-19), T cell activation (*P* = 7.18e-25), lymphocyte differentiation (*P* = 9.61e-19), mononuclear cell differentiation (*P* = 5.94e-18), regulation of T cell activation (*P* = 9.47e-15), etc*.* according to GO analysis (Supplementary Fig. 1A). In terms of KEGG pathway analysis, three significantly enriched pathways were hematopoietic cell lineage (*P* = 1.85e-14), Th1 and Th2 cell differentiation (*P* = 1.85e-14) and Th17 cell differentiation (*P* = 6.42e-13) (Supplementary Fig. 1B) [31–33]. These results strongly implied that T cells are actively engaged in the occurrence and development of sepsis.

### Immune-related DEGs involved in the progression of sepsis

**Fig. 4:**
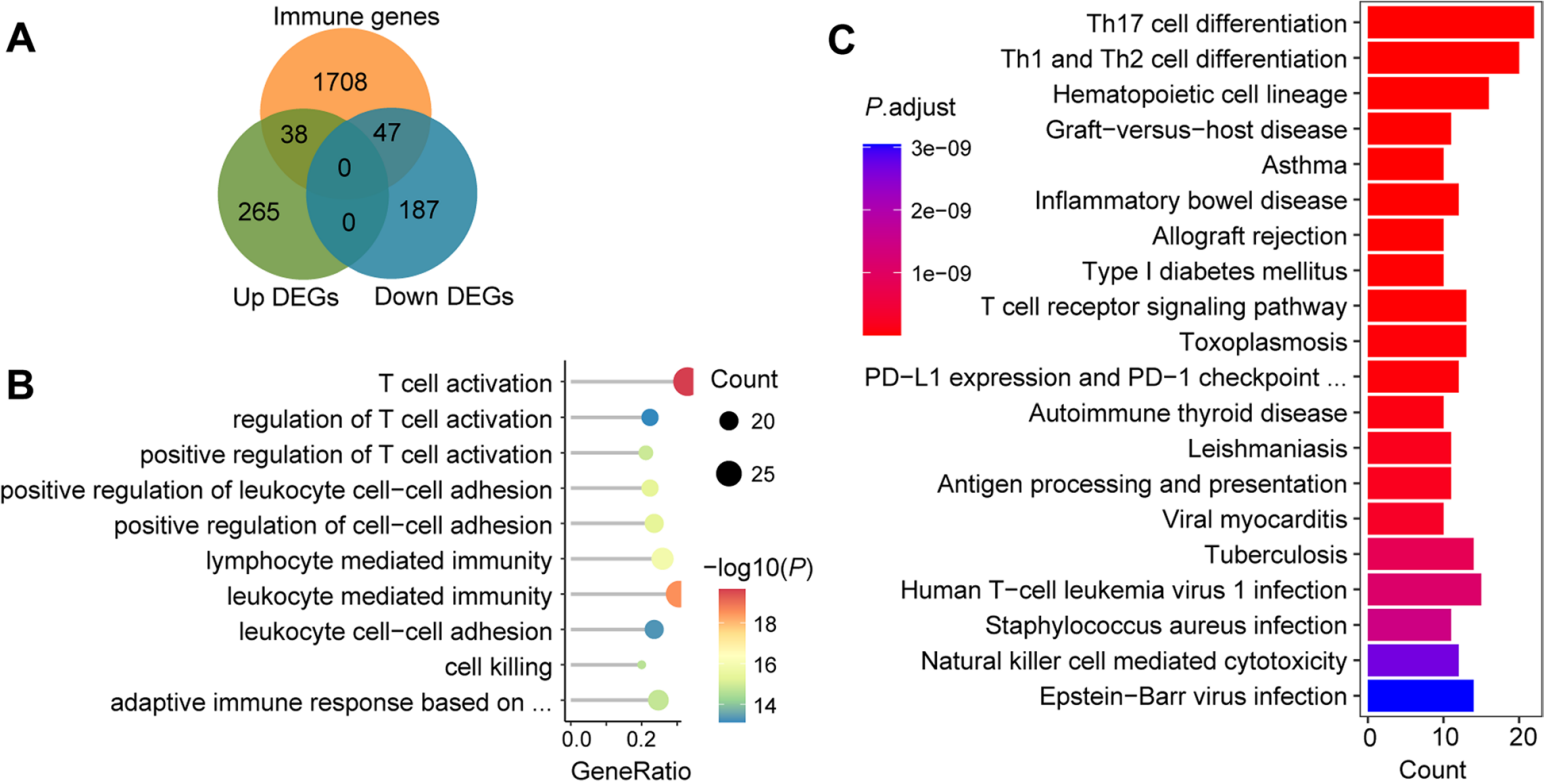
Venn diagram and enrichment analysis results of immune-related DEGs. **A** The Venn diagram showed 85 immune-related DEGs for common DEGs among three datasets, of which 38 are up-regulated, and 47 are down-regulated. **B** Top 10 GO terms in the biological process of immune-related DEGs. Low *p*-values are in orange red and high *p*-values are in green; the size of the circle is proportional to the number of enriched genes. **C** Top 20 KEGG pathways of immune-related DEGs. Low p-values are mentioned in red and high p-values are mentioned in blue

To identify the feature immune-related genes in sepsis, genes at the intersection between 537 common DEGs and immune-related gene set were analyzed. There were 85 immune-related DEGs (47 down-regulated and 38 up-regulated) (Fig. 4A). GO and KEGG enrichment analysis revealed that these genes were mainly associated with the biological processes of T cell activation (*P* = 1.53e-20), leukocyte mediated immunity (*P* = 2.99e-19), lymphocyte-mediated immunity (*P* = 1.41e-16), and pathways of Th1 and Th2 cell differentiation (*P* = 5.65e-26) and Th17 cell differentiation (*P* = 1.72e-22) (Fig. 4B, C) [31–33].

### PPI network analysis and submodule analysis

**Fig. 5:**
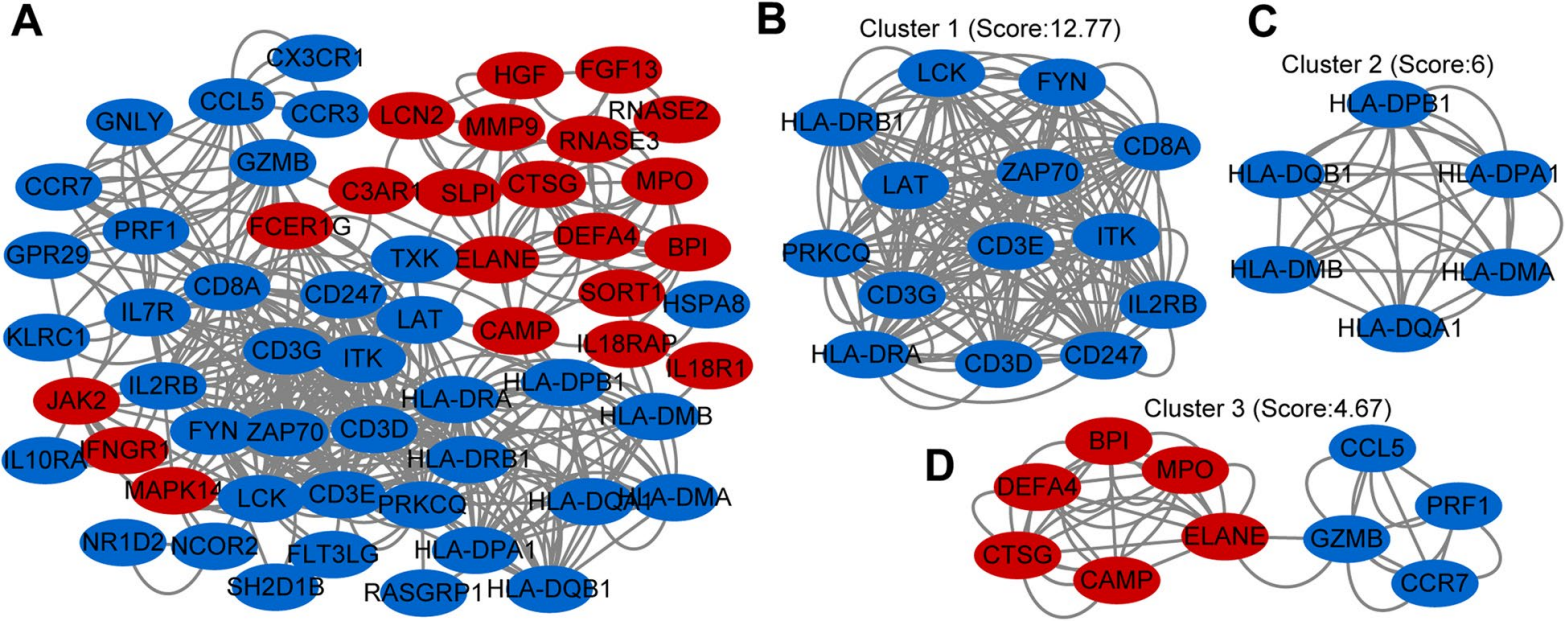
PPI network and three significant modules of immune-related DEGs. **A** PPI network of immune-related DEGs generated by STRING. Circles represent genes and lines represent PPIs. **B**-**D** The top three significant modules identified by MCODE scored 12.77, 6, and 4.67, respectively

PPI network allows the detection of the modules or hub genes associated with sepsis. A total of 85 overlapped genes were analyzed to characterize the potential protein–protein interaction. The PPIs with a confidence score ≥ 0.7 were selected and then imported into Cytoscape for further complex network analysis. The network contained 61 nodes and 408 interaction pairs (Fig. 5A). Three tightly connected gene submodules including 30 immune-related DEGs were obtained through the MCODE plugin of Cytoscape (Fig. 5B-D). GO analysis revealed that these genes were associated with T cell activation and cell–cell adhesion (Supplementary Fig. 2A). KEGG pathway enrichment analysis demonstrated that these genes primarily participated in Th1 and Th2 cell differentiation and Th17 cell differentiation (Supplementary Fig. 2B) [31–33].

### Identification and functional analysis of hub genes

**Fig. 6:**
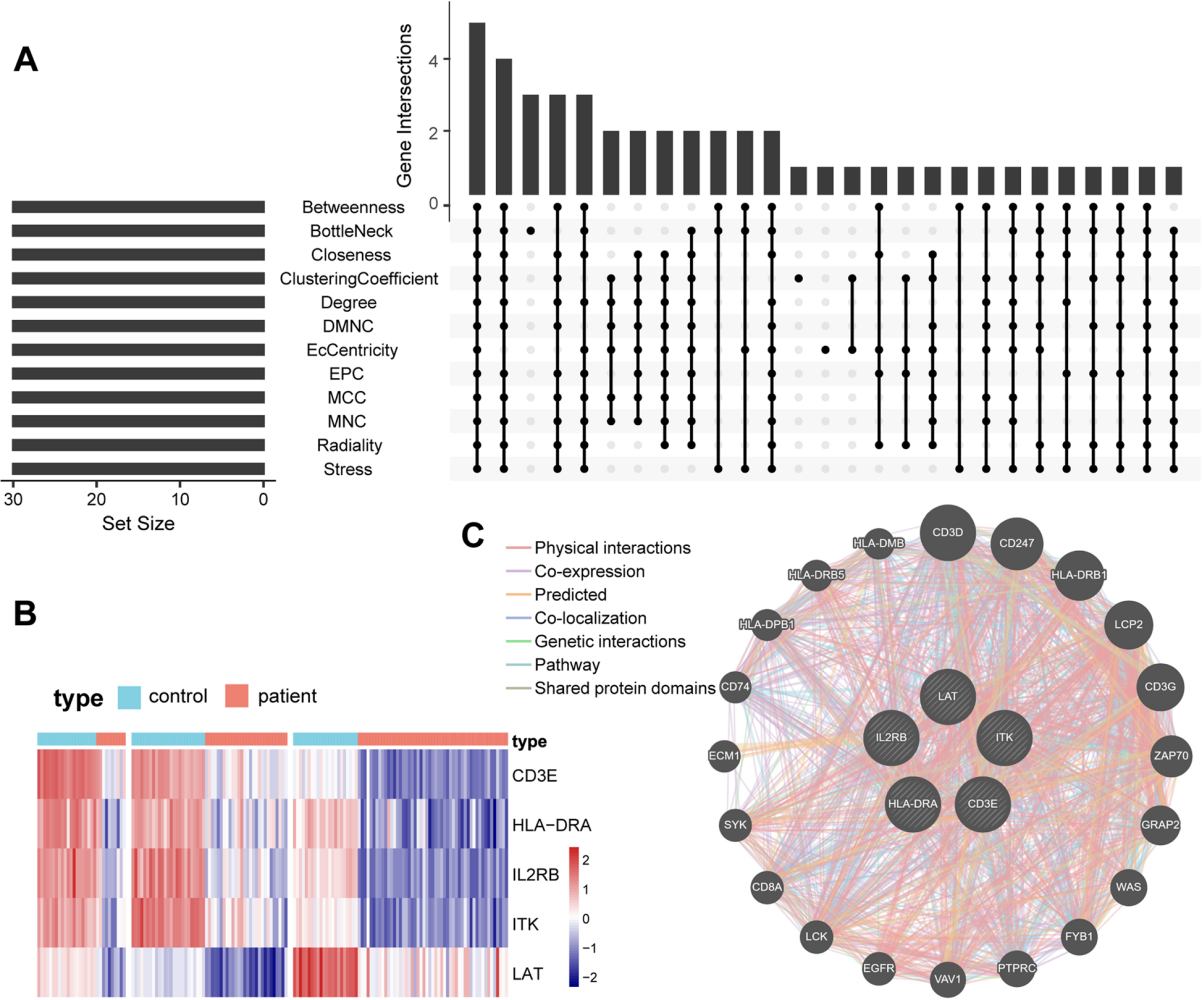
UpSet diagram, heatmap plot and co-expression network of hub genes. **A** UpSet diagram of 12 topological algorithms determined by PPI network analysis screened out five overlapping hub genes. **B** Expression patterns of hub genes between sepsis and healthy in each dataset. **C** GeneMANIA analyzed hub genes and their co-expression genes

To predict and explore the significant hub genes in the PPI network, cytoHubba with default parameters was used. The top 30 genes ranked by 12 different cytoHubba algorithms in the PPI network were intersected. Five hub genes, *CD3E*, *HLA-DRA*, *IL2RB*, *ITK* and *LAT*, were obtained for further exploration. The expression level of these five genes between sepsis and healthy controls has been summarized in a heatmap (Fig. 6A-B). The Wilcox test showed that the differences between sepsis and healthy controls in the three datasets were statistically significant. The details of these five hub genes were shown in Supplementary Table 3. The co-expression network of these genes was analyzed based on the GeneMANIA database. These genes displayed the complicated PPI network with physical interactions of 77.64%, co-expression of 8.01%, predicted of 5.37%, co-localization of 3.63%, genetic interactions of 2.87%, pathway of 1.88% and shared protein domains of 0.60% (Fig. 6C).

**Fig. 7:**
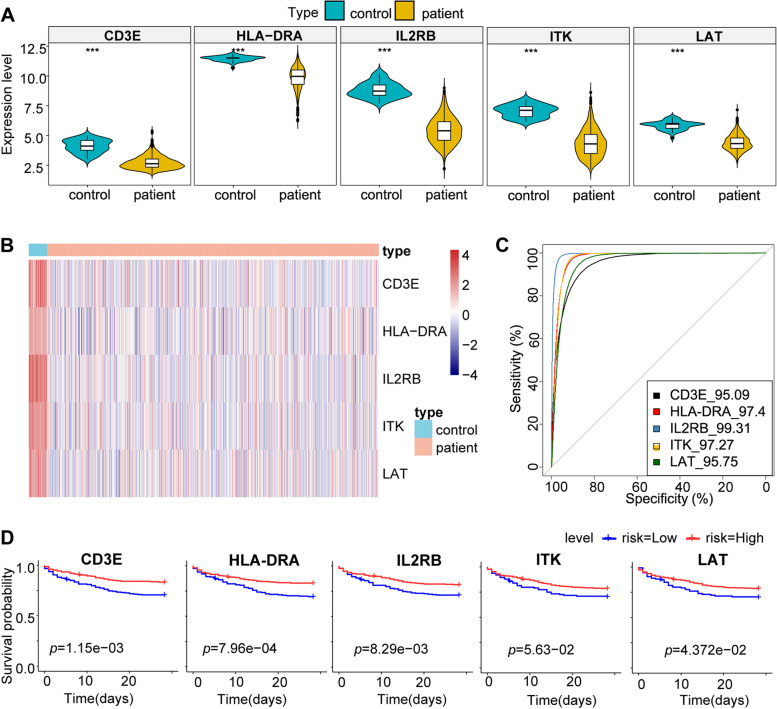
Validation of diagnostic hub genes. **A** Hub gene expression patterns in sepsis and healthy in GSE65682. **B** The heatmap shows the significantly different expression of hub genes between sepsis and healthy in GSE65682. **C** The ROC curve shows the diagnostic efficacy verification in GSE65682. **D** Kaplan–Meier survival curves by the expression level of hub genes. The sepsis patients were divided into high and low-expression groups based on the best cut-off value of gene expression

To verify the reliability of these five hub genes’ expression levels, we have utilized another dataset to analyze the expression levels. The results presented that all five hub genes were significantly down-regulated in sepsis patients compared with healthy controls (Fig. 7A, B). The five hub genes’ diagnostic efficacy was evaluated by plotting the ROC curves. In the GSE65682 dataset, *CD3E* (AUC:0.9509), *HLA-DRA* (AUC:0.974), *IL2RB* (AUC:0.9931), *ITK* (AUC:0.9727) and *LAT* (AUC:0.9575) showed good diagnostic efficiency in distinguishing sepsis patients from healthy controls (Fig. 7C). In addition, the Kaplan–Meier analysis showed that sepsis patients with lower expression of hub genes had a worse overall survival (Fig. 7D).

### Drugs from DrugBank

**Fig. 8:**
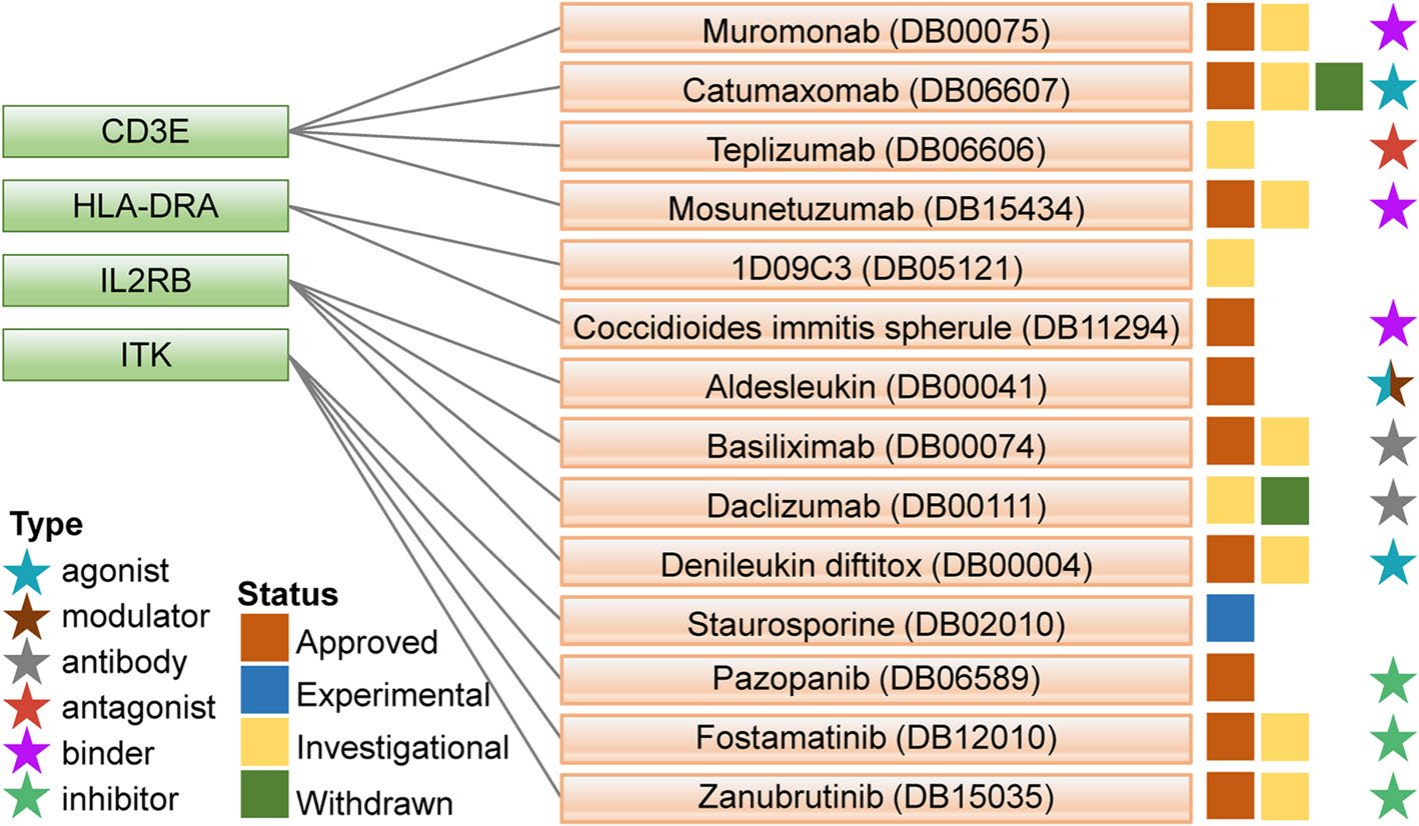
Drugs targeting five hub genes were extracted on the basis of the DrugBank database. Drug statuses, including approved, experimental, investigational and withdrawn, are indicated by colored squares. Drug types, including agonist, modulator, antibody, antagonist, binder and inhibitor, are indicated by colored starriness

A total of 14 drugs targeting five hub genes were obtained in reliance on drug and target information from the DrugBank database (Fig. 8). Of these, 10 drugs were approved, 10 were investigational drugs, one was experimental drug and two were withdrawn from the market.

Muromonab (DB00075) and Mosunetuzumab (DB15434) are binders of *CD3E*. Muromonab is an immunosuppressive therapy in kidney, heart, and liver transplant patients. While Mosunetuzumab is used to treat relapsed or refractory follicular lymphoma. Catumaxomab (DB06607) is an agonist of *CD3E* that facilitates the immune system-mediated destruction of cancer cells. Teplizumab (DB06606) targets *CD3E* being investigated for treating Type 1 diabetes (T1D). 1D09C3 (DB05121) targeting *HLA-DRA* is investigated for killing tumor cells by inducing programmed cell death. Coccidioides immitis spherule (DB11294) is a binder of *HLA-DRA* used to detect the late-onset hypersensitivity to Coccidioides immitis in individuals with a history of pulmonary coccidioidomycosis. Aldesleukin (DB00041) is not only an *IL2RB* agonist, but also a modulator of *IL2RB* which is used to induce the adaptive immune responses in renal cell carcinoma treatment. Basiliximab (DB00074) and Daclizumab (DB00111) are antibodies to *IL2RB*. Basiliximab is used as an immunosuppressive therapy in kidney transplant patients, whereas Daclizumab treats relapsed multiple sclerosis by blocking the interleukin-2 receptor. Denileukin diftitox (DB00004) is an agonist of *IL2RB* for the remedy of cutaneous T-cell lymphoma. Staurosporine (DB02010) is experimentally used to enhance the cAMP-mediated responses in human neuroblastoma cells. Pazopanib (DB06589), Fostamatinib (DB12010), and Zanubrutinib (DB15035) are inhibitors of *ITK*. Pazopanib is indicated for the treatment of advanced renal cell carcinoma and advanced soft tissue sarcoma in patients with prior chemotherapy. Fostamatinib is used to treat chronic immune thrombocytopenia after another treatment has been tried, and Zanubrutinib is used to treat mantle cell lymphoma.

### The potential therapeutic effects of catumaxomab and aldesleukin in sepsis

**Fig. 9:**
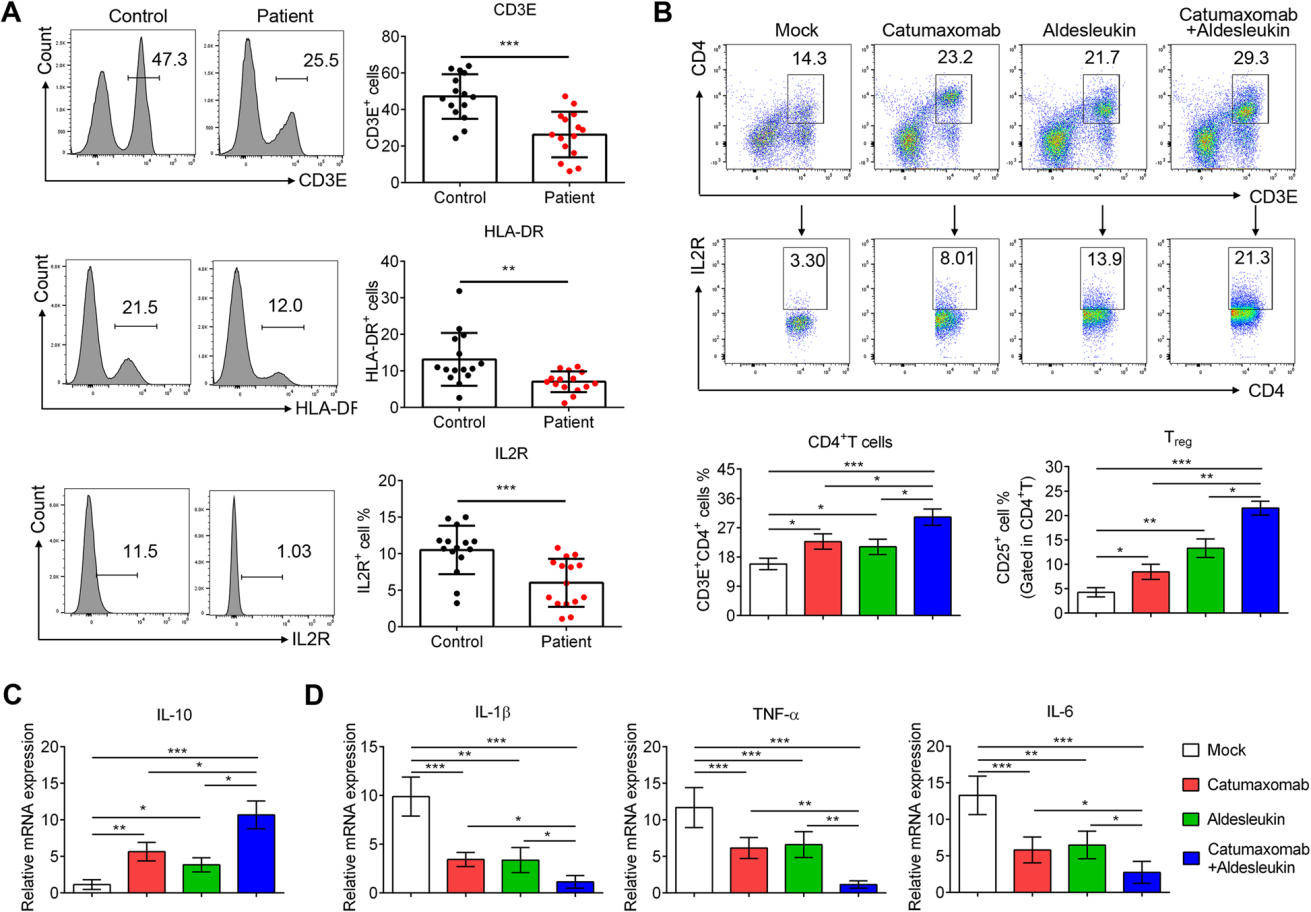
Catumaxomab and aldesleukin have the potential to inhibit sepsis. **A** The frequency of CD3E^+^ T cells, HLA-DR^+^T cells and IL-2R^+^ T cells in PBMC from patients with sepsis or healthy controls (*n* = 15). **B** The proportion of CD4^+^ T cells and CD4^+^ CD25^+^ T cells (gated in CD3E.^+^) after stimulated with catumaxomab (1 μg/ml) and aldesleukin (20 ng/ml) for 24 h (*n* = 5). *IL-10* (**C**), *IL-1*β, *TNF-*α and *IL-6* (**D**) expression level were measured by QPCR (*n* = 5)

PBMC samples were collected from 15 patients with sepsis and 15 healthy controls, and *CD3E*, *HLA-DR* and *IL-2R* were tested with flow cytometry. The protein expression of these molecular were significantly decreased in the peripheral blood of patients with sepsis (Fig. 9A). Based on target-based drug screen results, catumaxomab (*CD3E* agonist antibody) and aldesleukin (Recombinant protein of *IL-2R*) were chosen to examine the therapy effect. In vitro, the sepsis PBMCs were treated with catumaxomab and aldesleukin. After stimulation for 24 h, CD4^+^ T cell was significantly higher in the stimulants group than of the mock group (Fig. 9B). Furthermore, CD3E^+^CD4^+^IL2R^+^ cells, so-called regulatory T cells (Treg), were also increased with treatment. Treg functions as a protector in sepsis by secreting *IL-10* [40]. Thus, we also detected the expression levels of cytokines upon catumaxomab and aldesleukin treatments. As expected, *IL-10* was increased after T cell specific stimuli (Fig. 9C). While the expression of proinflammatory factors in sepsis (*IL-6*, *IL-1*β and *TNF-*α), which contributed to septic shock, were decreased (Fig. 9D). These results indicated that catumaxomab and aldesleukin treatment induced Treg cell activity and alleviated sepsis.

## Discussion

Sepsis is a clinical syndrome defined as "life-threatening organ dysfunction caused by a dysregulated host immune response to infection" [41]. The host response to sepsis is characterized by both pro-inflammatory responses and anti-inflammatory immune suppressive responses [42]. Sepsis clearly alters the innate and adaptive immune responses for sustained periods of time after clinical recovery, with immune suppression, chronic inflammation, and immune paralysis being common [43]. Deficits in the adaptive immune response may play a major role in sepsis patient mortality. The adaptive immune response involves a number of cell types including T cells, B cells, and dendritic cells, all with immunoregulatory roles aimed at limiting damage and returning immune homeostasis after infection [41, 44]. Our ability to discriminate adaptive and maladaptive immune responses in sepsis is limited. The dysregulated host immune response activated during sepsis may persist for up to 1 year [44]. Transcriptomic research turns out to be an effective tool for elucidating the molecular processes that regulate sepsis. This study aimed to identify significant genes and molecular dysregulation pathways associated with sepsis by applying bioinformatics analysis to sequencing data related to sepsis. Previous studies have limitations of the negligible amount of individuals in each study due to the high cost of these techniques, as well as the differences between different analysis and their platforms which are challenging to interpret and compare the results between different research groups. Three publicly accessible datasets were used in the analysis for this study. A total of 537 shared DEGs with similar expression patterns were discovered by identifying the overlapping DEGs that were present in all three datasets. One of these DEGs, *MCEMP1*, for instance, is highly expressed in sepsis, its down-regulation inhibited the inflammation of septic mice [45]. In our study, it was shown that sepsis cases had a noticeably higher expression of *CD177*. The finding aligns with earlier research that connected neutrophil transmigration and *CD177* to inflammatory diseases [46, 47]. It also protects the intestines from inflammation in IBD [46]. *S100A12* is highly increased during inflammation, which induces monocyte activation [48–50]. As mentioned in one literature review, *ANXA3* was also up-regulated in sepsis, but its role is unclear [51]. It may enhance the prolonged survival of neutrophils and pathogen clearance in the early phase, but result in organ failure at a later stage [51]. *GYG1* is an enzyme of glycogen synthesis, which was up-regulated in sepsis. Glycogen metabolism also regulated macrophage-mediated acute inflammatory responses [52]. Glycogen disorder is common in patients with severe sepsis [53].

This study demonstrated that different biological processes were significantly enriched in the DEGs, whether these genes were found in individual groups or were shared by several groups. T cell activation, leukocyte cell–cell adhesion, T cell differentiation, leukocyte differentiation, mononuclear cell differentiation, and pathways of hematopoietic cell lineage, as well as Th1, Th2 and Th17 cell differentiation were among these processes, but they were not limited to them. Furthermore, DEGs were also found to be enriched in pathways related to *Staphylococcus aureus* infection, among others [31–33]*.* This strongly suggests that T cells are involved in the occurrence and development of sepsis. The pathophysiology of T cell modifications may involve both intrinsic processes that directly affect T cells as well as indirect mechanisms that affect antigen-presenting cell or immature neutrophil activities, according to previous studies [54–57]. Th17 cells, a distinct subset of T helper (Th) cells recognized for their production of *IL-17*, have been strongly related to the onset and development of a number of inflammatory reactions and autoimmune diseases [58]. Additionally, the 28-day mortality among patients with severe sepsis and ICU-acquired infections have both been linked to a continually shifting Th2 / Th1 cell ratio [58]. Our findings strongly indicate that T cells, particularly Th1, Th2 and Th17, play a pivotal role in sepsis development.

In our study, the PPI network analysis and submodule analysis also suggested that these genes in most top submodules were also related to T cell activation, cell–cell adhesion as well as Th1, Th2 and Th17 cell differentiation. Moreover, we used the plug-in cytoHbba to disclose the essential hub genes in the PPI network. Five hub genes, *CD3E*, *HLA-DRA*, *IL2RB*, *ITK* and *LAT* were explored. *CD3E* forms the T-cell receptor-CD3 complex, which couples antigen recognition to intracellular signal transduction pathways and is down-regulated in sepsis [59]. Prior studies have highlighted the importance of *HLA-DRA* as a promising future biomarker for evaluating immunosuppression in sepsis [60]. *IL2RB* is a crucial mediator of Th1 and Th17 cellular immunity, which plays vital roles in the immune response against bacteria and fungi [61]. The expression level of *IL2RB* negatively correlates with mortality [61]. *ITK* is involved in regulating thermal homeostasis in mast cell responses in LPS-induced sepsis, and its lack leads to hypothermia exacerbation [62]. *LAT* is a crucial adaptor molecule in the TCR signaling pathway, and directly recruiting from cell surface *LAT* to microclusters is also critical for T-cell activation [63, 64]. The level of T cell activation may be influenced by the quantity of *LAT* on the cell surface [64, 65]. The T cell dysfunction may cause immunosuppression after acute sepsis [1]. Furthermore, KM analysis showed that sepsis with high expression values of these five hub genes had a better prognosis, suggesting that these five hub genes may serve as important therapeutic targets or biomarkers for sepsis. Our preliminary experiment found that *CD3E*, *IL2R* and *HLA-DR* were significantly reduced in sepsis when compared to healthy control.

Although mortality rates have improved, new drugs for sepsis are still required. Fourteen drugs targeting the above immune-related genes were obtained. Many of these compounds were previously approved and used as immunosuppressants or used to treat the diseases of immune cells in addition to cancer. Muromonab CD3, the treatment of acute solid organ transplant rejection, has proven an effective alternative and gives a substantial new perspective on immunosuppressive therapy [66]. Anti-CD20/CD3 T-cell dependent bispecific (TDB) antibody mosunetuzumab is entirely humanized full-length and assembled utilizing the knobs-into-holes technology [67, 68]. Mosunetuzumab and blinatumomab share a similar mode of action: B-cell lysis and T-cell activation result from mosunetuzumab's dual binding to CD20 on malignant B-cells and CD3 on T-cells [69]. Catumaxomab increases the activation of immune cells by combining the cancer cells, T cells, and auxiliary immune cells into proximity. It makes it easier for the immune system to kill cancer cells [70]. Teplizumab alters CD8^+^ T lymphocytes, which are believed to be the most critical effector cells that kill beta cells [71]. Interleukin-2 (Aldesleukin) has been licensed by the Food and Drug Administration (FDA) for the treatment of individuals with advanced forms of renal cell carcinoma (metastatic RCC) and melanoma [72]. Basiliximab or daclizumab in combination with triple treatment was an effective and safe immunosuppressive strategy, as indicated by a low incidence of acute rejections, excellent graft function, high survival rates, and an acceptable adverse event profile in adult patients one year after deceased donor renal transplantation [73]. A recombinant fusion protein called denileukin diftitox treats the cutaneous T cell lymphomas that express IL-2 receptors [57]. Human interleukin-2 (IL-2) is linked to diphtheria toxin fragments A and B [57]. Three out of 14 were BTK and ITK inhibitors such as Pazopanib, Fostamatinib and Zanubrutinib. Cytoplasmic tyrosine kinases BTK and ITK are essential for forming B and T cells, and loss-of-function mutations in either result in X-linked agammaglobulinemia and an increased risk of a severe, usually fatal Epstein-Barr virus infection, respectively [74]. Pazopanib, an approved medication for handling renal cell carcinoma and soft tissue sarcoma, is a VEGF receptor, platelet-derived growth factor receptor, fibroblast growth factor receptor, and stem cell receptor c-Kit inhibitor [75]. The abovementioned drugs are relevant to the immune balance in other diseases and may benefit sepsis patients. For example, we demonstrated in this study that catumaxomab and aldesleukin (agonists targeting CD3E and IL2R separately) effectively restore T cells' regulatory activity and suppress excessive inflammation, which is critical in reducing the occurrence of septic shock. At present, antibiotic treatment of sepsis is facing the problem of microbial resistance. Our research is based on drug targets for host immune regulation that do not develop antimicrobial resistance and have better application prospects than antibiotics. However, future studies based on the investigations of in vitro or animal models will be necessary to confirm these possibilities.

## Conclusion

In conclusion, sepsis is a complicated clinical disease characterized by dysregulated immune responses that can linger long after the initial recovery. Immunological suppression, persistent inflammation, and immunological paralysis are frequently caused by this immune dysregulation, which may be a factor in patient death. The study used bioinformatics analysis and transcriptome research to investigate the complex biological mechanisms involved in sepsis. Analyzing publicly available data sets, the study identified 537 differentially expressed genes (DEGs) with similar patterns, revealing significant sepsis-related genes such as *MCEMP1*, *CD177*, *S100A12*, *ANXA3*, and *GYG1*. Furthermore, the study also showed a notable enrichment of biological pathways and processes involved in T cell activation, leukocyte adhesion, differentiation, and immunological responses, including Th1, Th2, and Th17 cell differentiation. This highlights the critical part T cells play in the onset and progression of sepsis, as well as their potential as biomarkers. The protein–protein interaction network analysis revealed hub genes, including *CD3E*, *HLA-DRA*, *IL2RB*, *ITK*, and *LAT*, which are all involved in T cell activation and immune regulation. Better outcomes for sepsis patients were associated with high expression of these hub genes. In addition, the study investigated potential drug candidates that target immune-related genes, some of which have demonstrated promise in immunosuppression and cancer treatment. These medications have the potential to treat sepsis, but more studies, especially those using in vitro and animal models, are required to validate their effectiveness.

### Supplementary Information

**Additional file 1:** **Figure S1.** Enrichment analysis results of common DEGs among three datasets (GSE28750, GSE57065 and GSE95233). (A) Top 10 GO term in biological process for common DEGs. (B) Top 20 KEGG pathway for common DEGs. **Figure S2.** Enrichment analysis results of immune-related DEGs from PPI submodules. (A) Top 10 GO term in biological process for DEGs. (B) Top 20 KEGG pathway for DEGs.

**Additional file 2:** **Table S1.** The list of immune-related genes.

**Additional file 3:** **Table S2.** The top significant DEGs among three datasets.

**Additional file 4:** **Table S3.** The detailed five hub genes.

## Data Availability

The datasets supporting this study are available from GEO database. And corresponding code of this study can be found at https://github.com/liangpingping/BM_code, further inquiries can be directed to the corresponding authors.

## Abbreviations

DEG: Differentially expressed gene
GEO: Gene expression omnibus
GO: Gene ontology
KEGG: Kyoto encyclopedia of genes and genomes
PPI: Protein–protein interaction
KM: Kaplan–meier
ROC curve: Receiver operating characteristic curve
AUC: The area under the curve
