# Supplementary material for: Exploring the biomarkers and potential therapeutic drugs for sepsis via integrated bioinformatic analysis
Source: BMC Infect Dis. 2024 Jan 2;24:32. doi: 10.1186/s12879-023-08883-9 (PMC10763157; doi:10.1186/s12879-023-08883-9)
Supplement: Supplementary file 1 — Additional file 1: Figure S1. Enrichment analysis results of common DEGs among three datasets (GSE28750, GSE57065 and GSE95233). (A) Top 10 GO term in biological process for common DEGs. (B) Top 20 KEGG pathway for common DEGs. Figure S2. Enrichment analysis results of immune-related DEGs from PPI submodules. (A) Top 10 GO term in biological process for DEGs. (B) Top 20 KEGG pathway for DEGs. [file 12879_2023_8883_MOESM1_ESM.docx]

**Supplementary Figures**

**Exploring the biomarkers and potential therapeutic drugs for sepsis via integrated bioinformatic analysis**

Pingping Liang^1,^ ^2, #^, Yongjian Wu^2, #^, Siying Qu^3, #^, Muhammad Younis^1,^ ^2^, Wei Wang^1^, Zhilong Wu^1, *^ and Xi Huang^1, 2, *^

^1^ Foshan Fourth People’s Hospital, Foshan, Guangdong Province, 528041, China

^2^ Center for Infection and Immunity and Guangdong Provincial Engineering Research Center of Molecular Imaging, the Fifth Affiliated Hospital of Sun Yat-sen University, Zhuhai, Guangdong Province, 519000, China.

^3^ Department of Clinical Laboratory, Zhuhai Hospital of Integrated Traditional Chinese and Western Medicine, The Second People's Hospital of Zhuhai, Zhuhai, Guangdong, 519020, China

^#^ These authors contribute equally to this work.

***Correspondence:**Xi Huang, Email: [huangxi6@mail.sysu.edu.cn](mailto:huangxi6@mail.sysu.edu.cn)


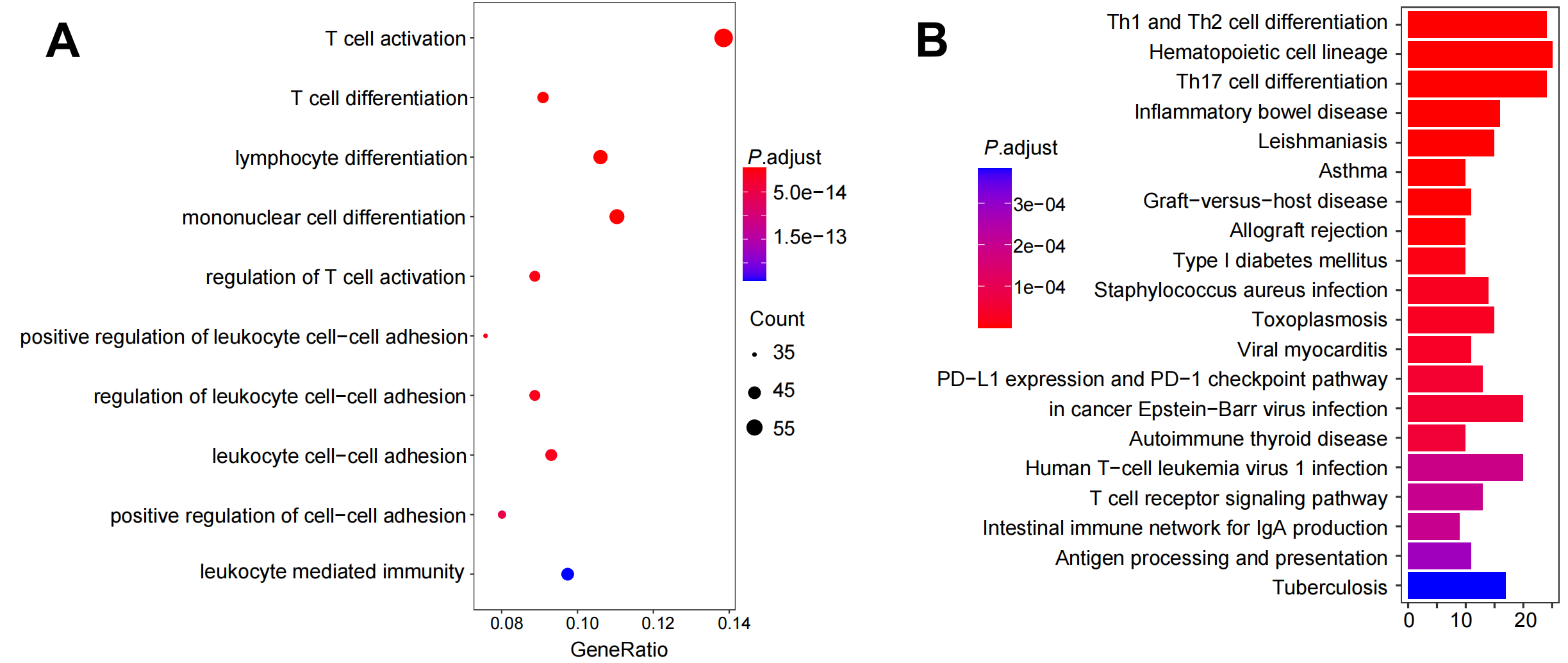


**Supplementary Figure 1. Enrichment analysis results of common DEGs among three datasets (GSE28750, GSE57065 and GSE95233). (A)** Top 10 GO term in biological process for common DEGs. **(B)** Top 20 KEGG pathway for common DEGs.


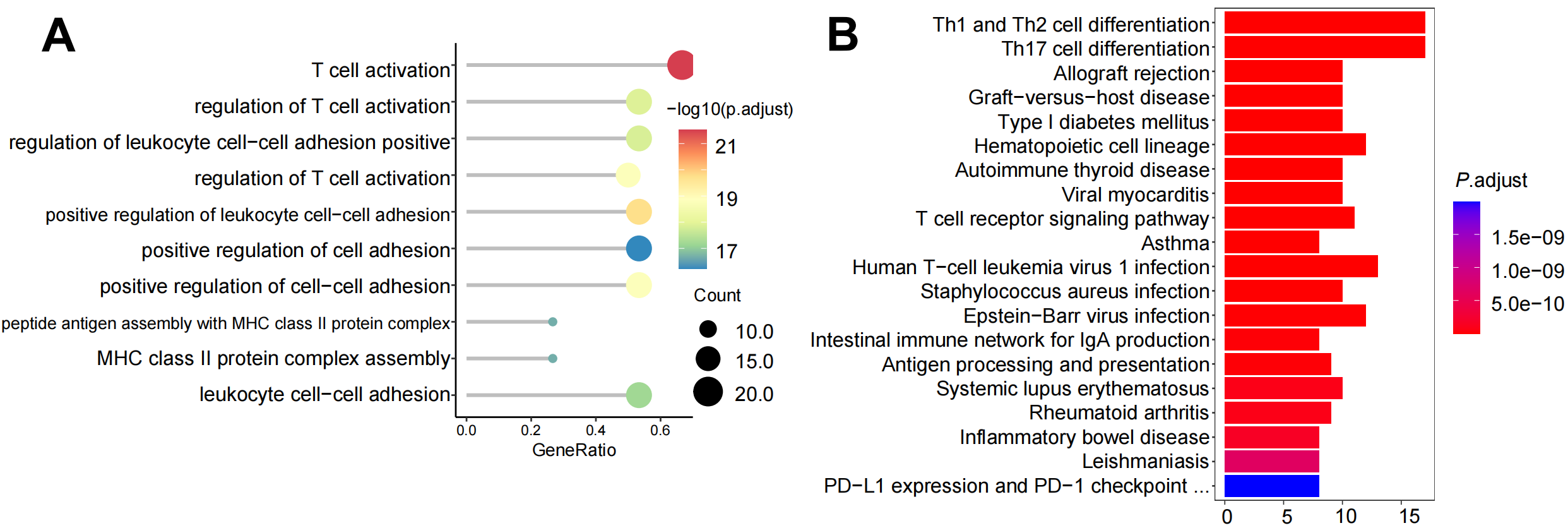


**Supplementary Figure 2. Enrichment analysis results of immune-related DEGs from PPI submodules. (A)** Top 10 GO term in biological process for DEGs. **(B)** Top 20 KEGG pathway for DEGs.
